# Short Chain Fatty Acids Enhance Aryl Hydrocarbon (Ah) Responsiveness in Mouse Colonocytes and Caco-2 Human Colon Cancer Cells

**DOI:** 10.1038/s41598-017-10824-x

**Published:** 2017-08-31

**Authors:** Un-Ho Jin, Yating Cheng, Hyejin Park, Laurie A. Davidson, Evelyn S. Callaway, Robert S. Chapkin, Arul Jayaraman, Andrew Asante, Clinton Allred, Evelyn A. Weaver, Stephen Safe

**Affiliations:** 1 Department of Veterinary Physiology and Pharmacology, Texas A&M University, College Station, TX 77843 USA; 2 Department of Nutrition and Food Science, Texas A&M University, College Station, TX 77843 USA; 3 Artie McFerrin Department of Chemical Engineering, Texas A&M University, College Station, TX 77843 USA; 4 Department of Biology, Alabama State University, Montgomery, AL 36101 USA; 5 Department of Animal Science, Texas A&M University, College Station, TX 77843 USA

## Abstract

Aryl hydrocarbon receptor (AhR) ligands are important for gastrointestinal health and play a role in gut inflammation and the induction of T regulatory cells, and the short chain fatty acids (SCFAs) butyrate, propionate and acetate also induce similar protective responses. Initial studies with butyrate demonstrated that this compound significantly increased expression of Ah-responsive genes such as *Cyp1a1/CYP1A1* in YAMC mouse colonocytes and Caco-2 human colon cancer cell lines. Butyrate synergistically enhanced AhR ligand-induced Cyp1a1/CYP1A1 in these cells with comparable enhancement being observed for 2,3,7,8-tetrachlorodibenzo-*p*-dioxin (TCDD) and also microbiota-derived AhR ligands tryptamine, indole and 1,4-dihydroxy-2-naphthoic acid (DHNA). The effects of butyrate on enhancing induction of Cyp1b1/CYP1B1, AhR repressor (Ahrr/AhRR) and TCDD-inducible poly(ADP-ribose)polymerase (Tiparp/TiPARP) by AhR ligands were gene- and cell context-dependent with the Caco-2 cells being the most responsive cell line. Like butyrate and propionate, the prototypical hydroxyamic acid-derived histone deacetylase (HDAC) inhibitors Panobinostat and Vorinostat also enhanced AhR ligand-mediated induction and this was accompanied by enhanced histone acetylation. Acetate also enhanced basal and ligand-inducible Ah responsiveness and histone acetylation, demonstrating that acetate was an HDAC inhibitor. These results demonstrate SCFA-AhR ligand interactions in YAMC and Caco-2 cells where SCFAs synergistically enhance basal and ligand-induced expression of AhR-responsive genes.

## Introduction

The aryl hydrocarbon receptor (AhR) was initially identified as the hepatic intracellular protein that bound with high affinity to 2,3,7,8-tetrachlorodibenzo-*p*-dioxin (TCDD), a highly toxic industrial by-product^1^. The AhR partners with the AhR nuclear translocator (Arnt) to form a nuclear heterodimer that binds with *cis*-acting xenobiotic response element (XREs) in target gene promoters to induce expression of Ah-responsive genes such as *CYP1A1* ^2, 3^. Although the AhR is an essential target for mediating the toxicity of TCDD and structurally-related compounds^4, 5^, there is increasing evidence that the AhR plays an essential role in maintaining tissue-specific homeostasis^6–9^. For example, in AhR knockout mice, there are multiple deficits including reproductive tract and cardiovascular problems and failure of closure of the ductis venosis in liver^6–9^. The AhR also plays an important role in regulating the immune functions, inflammation, gastrointestinal health, stem cells and cancer, and this receptor is now emerging as an important drug target [rev. in refs 10–15].

TCDD, structurally-related halogenated aromatics, and carcinogenic polynuclear aromatic hydrocarbons were the first compounds identified as AhR ligands; however, subsequent studies have identified structurally diverse synthetic and naturally occurring compounds as ligands for this receptor. These include industrial by-products, phytochemicals including flavonoids, indole-3-carbinol and related indole compounds, diverse pharmaceuticals, and possible endogenous ligands including formylindolino[2,3]b-carbazol (FICZ) [rev. in refs 15 and 16]. The identification of health-promoting compounds as AhR ligands has spurred research on the development and applications of AhR-active chemicals for chemotherapeutic applications.

The AhR plays a particularly important role in maintaining gastrointestinal health and several studies show that AhR expression in subsets of gut epithelial cells are important for bacterial resistance, gut inflammation and integrity and this has been associated with induction of T regulatory (Treg) cells, FoxP3 and interleukin-22^17–25^. Moreover, these same studies show that AhR-active microbiota-derived metabolites including tryptophan catabolites or exogenous AhR-active compounds play a role in maintaining intestinal integrity and bacterial resistance and inhibiting inflammation. Interestingly, short chain fatty acids (SCFAs) such as butyrate exhibit activities similar to that observed for AhR agonists including induction of Treg cells, anti-inflammatory activity and induction of interleukin 22^26–29^. Since microbiota simultaneously produce both AhR ligands and SCFAs, we used human Caco-2 anticancer cells and mouse YMAC colonocytes as models to investigate the interaction of SCFAs and AhR ligands and their effects on Ah-responsive gene expression. The results demonstrate that acetate, propionate and butyrate enhance AhR ligand-induced responses via their activities as histone deacetylase (HDAC) inhibitors; however, the effectiveness of these interactions are both gene- and cell context-dependent. Our data also show for the first time that one of the most abundant SCFAs, acetate, is also in HDAC inhibitor.

## Materials and Methods

### Cell lines, antibodies, and reagents

The young adult mouse colonic (YAMC) cell line and the AhR knockout cells (YAMC-AhR-KO) were described in our previous studies^30^. Cells were maintained in RPMI 1640 medium with 5% fetal bovine serum, 5 units/ml mouse interferon-γ (IF005) (EMD Millipore, MA), 0.1% ITS “−” minus (insulin, transferrin, selenium) (41-400-045) (Life Technologies, Grand Island, NY) at 33 °C (permissive conditions) and experiments were carried out at 37 °C (nonpermissive conditions). Caco-2 human colon cancer cells were obtained from the American Type Culture Collection (ATCC, Manassas, VA) and maintained in Dulbecco’s modified Eagle’s medium (DMEM) nutrient mixture supplemented with 20% fetal bovine serum (FBS), 10 ml/L 100X MEM non-essential amino acid solution (Gibco), and 10 ml/L 100X antibiotic/antimycotic solution (Sigma-Aldrich, St. Louis, MO) at 37 °C in the presence of 5% CO_2_. β-Actin antibody was purchased from Sigma-Aldrich, and mouse CYP1A1 antibody was kindly provided by the late Dr. Paul Thomas (Rutgers University) and Dr. B. Moorthy (Baylor College of Medicine, Houston). Antibodies to human CYP1A1 and AhR were purchased from Santa Cruz Biotechnology (Santa Cruz, CA). Acetyl-H3K9/K14, acetyl-H3K27, and acetyl-H4K8 antibodies were purchased from Cell Signaling Technology (Danvers, MA). Sodium butyrate, sodium propionate, and sodium acetate were purchased from Sigma-Aldrich, and Panobinstat, Entinostat, Vorinostat were purchased from LC Laboratories (Woburn, MA). CH223191 was purchased from TOCRIS Bioscience (Minneapolis, MN).

### Chromatin immunoprecipitation (ChIP) assay

The ChIP assay was performed using ChIP-IT Express Magnetic Chromatin Immunoprecipitation kit (Active Motif, Carlsbad, CA) according to the manufacturer’s protocol. YAMC and Caco-2 cells (1 × 10^7^ cells) were treated with sodium butyrate overnight; 10 nM TCDD was added and after 2 hr, cells were fixed with 1% formaldehyde, and the cross-linking reaction was stopped by addition of 0.125 M glycine. After washing twice with phosphate-buffered saline, cells were scraped and pelleted. Collected cells were hypotonically lysed, and nuclei were collected. Nuclei were then sonicated to desired chromatin length (~200–1500-bp). The sonicated chromatin was immunoprecipitated with primary antibodies and protein A-conjugated magnetic beads at 4 °C for 8 hr. The magnetic beads were extensively washed, protein-DNA crosslinks were reversed and eluted, and DNA was prepared by proteinase K digestion followed by PCR amplification. The mouse *Cyp1a1* primers were 5′-CAG GAG AGC TGG CCC TTT A-3′ (sense) and 5′-TAA GCC TGC TC ATC CTG TG-3′ (antisense), and subsequently amplified by targeting a 215-bp region of mouse *Cyp1a1* promoter, which contained AhR-binding sequences. The human *CYP1A1* primers were 5′-TCA ATC AAG AGG CGC GAA CCT C-3′ (sense), and 5′-CTA CAG CCT ACC AGG ACT CG-3′ (antisense), and then amplified a 203-bp region of human *CYP1A1* promoter which contained the AhR binding sequences. PCR products were resolved on a 2% agarose gel in the presence of ethidium bromide.

### Quantitative real-time PCR

cDNA was prepared from the total RNA of cells using High Capacity RNA-to-cDNA Kit (Applied Biosystems, Foster City, CA). Each PCR was carried out in triplicate in a 20 μL volume using SYBR Green Q-PCR Master mix (GenDEPOT, Katy, TX) for 1 min at 95 °C for initial denaturing, followed by 40 cycles of 95 °C for 15 sec and 60 °C for 1 min in the Bio-Rad iCycler (MyiQ™2) real-time PCR System. The comparative CT method was used for relative quantitation of samples. Values for each gene were normalized to expression levels of TATA-binding protein (TBP). The sequences of the primers used for real-time PCR are summarized in Supplementary Table S1.

### Western blot analysis

Cells (3 × 10^5^) were plated in six-well plates in DMEM media containing 2.5% FBS for 24 hr and then treated with different concentrations of the compounds. Cell lysates were prepared in lysis buffer containing 50 mM HEPES, 0.5 M NaCl, 1.5 mM MgCl_2_, 1 mM EGTA, 10% glycerol, and 1% Triton-X-100, each 10 μL/ml protease and phosphatase inhibitor cocktail (GenDEPOT) and 1% NP-40. The cells were disrupted and extracted at 4 °C for 30 min and after centrifugation, the supernatant was obtained as the cell lysate. Protein concentrations were measured using the Bio-Rad protein assay. Aliquots of cellular proteins were electrophoresed on 10% SDS–polyacrylamide gel electrophoresis (PAGE) and transferred to a PVDF membrane (Bio-Rad, Hercules, CA). The membrane was allowed to react with a specific antibody, and detection of specific proteins was carried out by enhanced chemiluminescence. Loading differences were normalized using a polyclonal β-actin antibody.

### Animals and compounds administration

Mice (C57BL6/J) were housed in the Texas A&M University animal facility with a 12-hr light/dark cycle and constant temperature (23–25 °C). The mice had free access to water and diet. All procedures were performed in accordance with National Institutes of Health guidelines for the care and use of animals and were approved by the institutional animal care and use committee at Texas A&M University. For experiments involving butyrate and/or DHNA treatment, mice (8–10 weeks of age) were gavaged once per day with butyrate (1 g/kg in water) and/or 1,4-dihydroxy 2-naphthoic acid (DHNA, 20 mg/kg in water) for 3 days and killed 6 hr after the last treatment.

### Statistics

All of the experiments were repeated a minimum of three times. The data are expressed as the means ± SD. Statistical significance was analyzed using either Unpaired-Student’s t-test (two-tailed) or analysis of variance (ANOVA) test. A *P* value of less than 0.05 was considered statistically significant.

## Results

### Butyrate enhances basal and TCDD-induced Ah-responsive gene expression

**Figure 1:**
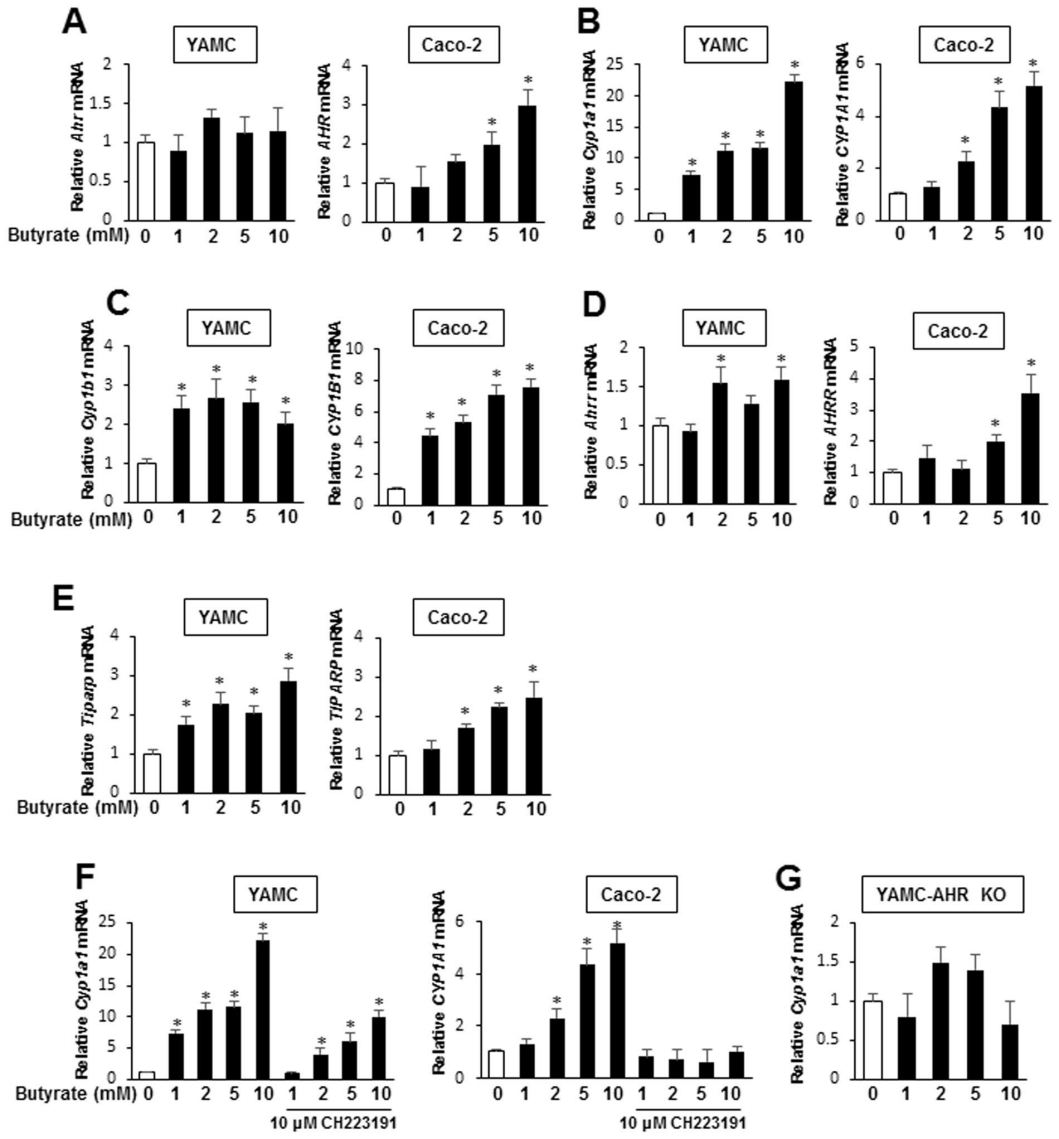
Butyrate modulates expression of Ah-responsive genes in YAMC and Caco-2 cells. Cells were treated with DMSO or 1–10 mM butyrate for 24 hr, and expression of *AhR* mRNA (**A**) and protein (**B**) were determined by real time PCR and western blots, respectively. Cells were treated with DMSO and 1–10 mM butyrate for 24 hr, and levels of *Cyp1a1/CYP1A1* (**C**), *Cyp1b1/CYP1B1* (**D**), *Ahrr/AhRR* (**D**) and *Tiparp/TiPARP* (**F**) mRNAs were determined by real time PCR. (**G**) Cells were treated with butyrate alone or in combination with CH223191, and *Cyp1a1/CYP1A1* mRNA was determined by real time PCR. (**H**) YAMC-AhR-KO cells were treated with butyrate, and *Cyp1a1* mRNA levels were determined by real time PCR. Results are expressed as means ± SE (3 replicated determination), and significantly (p < 0.05) induced (*) and inhibited (**) responses are indicated.

**Figure 2:**
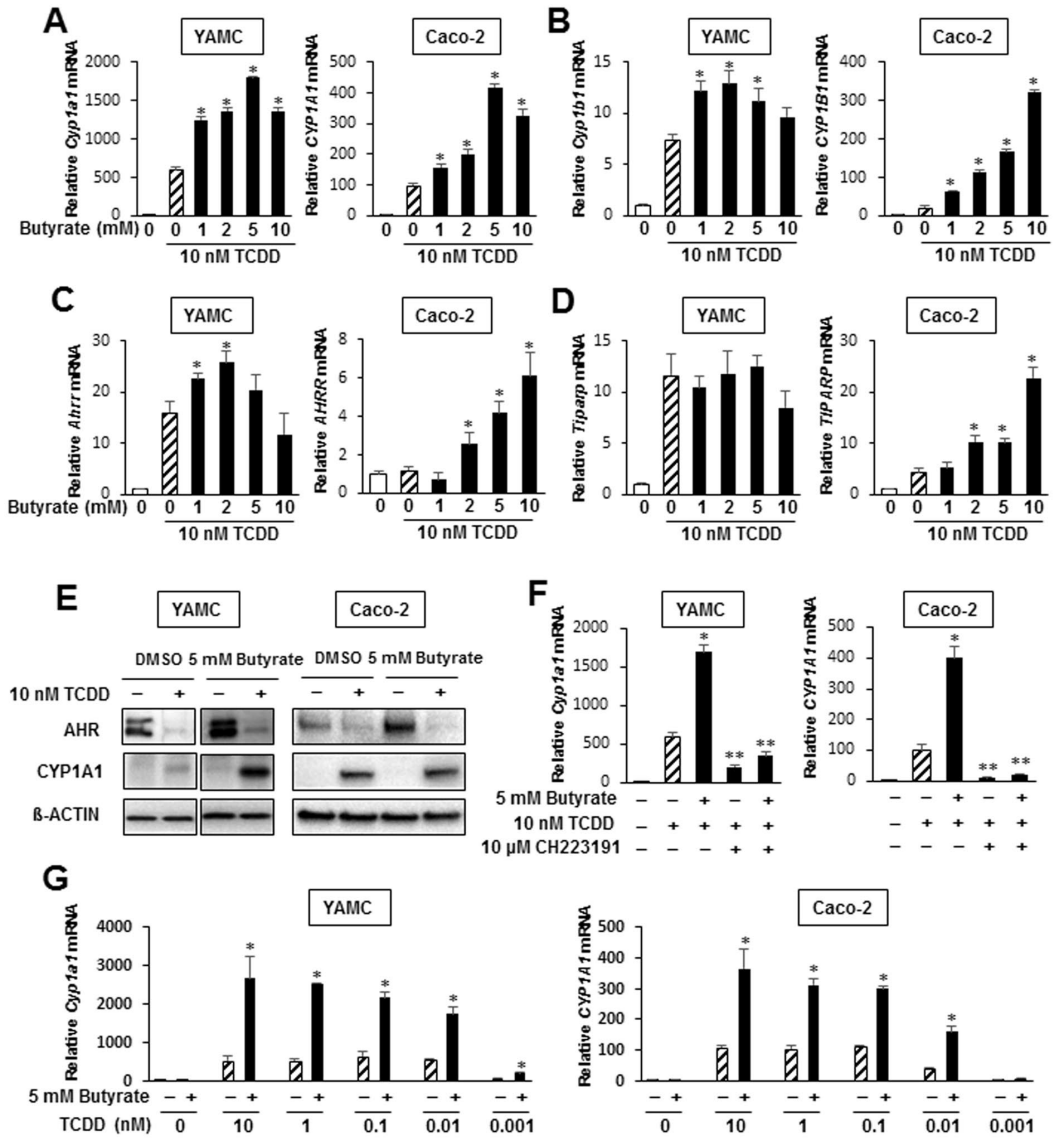
Butyrate enhances TCDD-induced gene expression YAMC and Caco-2 cells. Cells were treated with DMSO, TCDD and TCDD plus 5 mM butyrate, and effects on *Cyp1a1/CYP1A1* (**A**), *Cyp1b1/CYP1B1* (**B**), *Ahrr/AhRR* (**C**) and *Tiparp/TiPARP* (**D**) mRNA levels were determined by real time PCR. (**E**) Cells were treated with DMSO, TCDD, butyrate and their combination for 24 hr, and whole cell lysates were analyzed by western blots. (**F**) Cells were treated with DMSO, butyrate, TCDD and TCDD plus butyrate and also in combination with CH223191, and *Cyp1a1/CYP1A1* mRNA levels were determined by real time PCR. (**G**) Cells were treated with DMSO, 5 mM butyrate, different concentrations of TCDD alone and TCDD plus butyrate, and *Cyp1a1/CYP1A1* mRNA levels were determined by real time PCR. With the exception of *AhRR* in Caco-2 cells, TCDD significantly induced all other Ah-responsive genes, and significant (p < 0.05) enhancement by butyrate is indicated (*). Results are expressed as means ± SE for at least 3 separate determinations for each treatment group. Significant (p < 0.05) inhibition by CH223191 is indicated (**).

Sodium butyrate is a major microbiota-derived metabolite and potent HDAC inhibitor and there are conflicting reports showing that butyrate enhances^31^ or does not affect^32^ basal or AhR ligand-induced CYP1A1/CYP1A1-promoter activity. Treatment of YAMC and Caco-2 cells with 1–10 mM butyrate had minimal effects on *AhR* mRNA levels in YAMC cells but increased expression in Caco-2 cells (Fig. 1A). Butyrate alone induced two Ah-responsive genes, *Cyp1a1/CYP1A1* (Fig. 1B) and *Cyp1b1/CYP1B1* (Fig. 1C), in both YAMC and Caco-2 cells and the fold and maximal induction responses were generally higher for *Cyp1a1/CYP1A1*. Butyrate also significantly induced *AhRR* and *Tiparp* gene expression in both cell lines (Fig. 1D and E) and there was some cell context- and concentration-dependent variability in these responses. Using *Cyp1a1/CYP1A1* as a model, butyrate-induced gene expression was inhibited by the AhR antagonist CH223191 (Fig. 1F) and we observed that butyrate induction of *Cyp1a1* was also blocked in YAMC-AhR-KO cells (Fig. 1G) in which the AhR was knocked out via CRISPR/Cas9 as described^30^. Thus, butyrate induces Ah-responsive genes and this response is AhR-dependent; however, as indicated in subsequent studies (Fig. 2), the magnitude of the Cyp1a1 response was >5% of the induction response observed for TCDD.

We also investigated the effects of butyrate on TCDD-induced gene expression in YAMC and Caco-2 cells and Fig. 2A illustrates that butyrate enhanced TCDD-induced *Cyp1a1/CYP1A1* gene expression in YAMC and Caco-2 cells approximately 3- to 4-fold. However, the fold enhancement of other Ah-responsive genes was both cell context- and response-dependent. In YAMC cells, induction of Cyp1b1/CYP1B1 (Fig. 2B), AhRR (Fig. 2C) and TiPARP (Fig. 2D) by TCDD was minimally or not enhanced after cotreatment with butyrate, whereas highly significant enhancement was observed for all genes in Caco-2 cells. Interestingly, AhRR was not induced by TCDD in Caco-2 cells but in combination with butyrate, there was a >6-fold induction response. TCDD (10 nM) alone or in combination with 5 mM butyrate induced expression of Cyp1a1 protein and butyrate significantly enhanced this response in YAMC cells, whereas minimal enhancement was observed in Caco-2 cells (Fig. 2E). Butyrate increased expression of AhR protein in both cell lines and TCDD alone or in combination with butyrate decreased expression of AhR protein. This effect is commonly observed in most cell lines due to proteasome-dependent degradation of the AhR. Results in Fig. 2F show that the AhR antagonist CH223191 inhibited both TCDD- and TCDD plus butyrate-induced Cyp1a1/CYP1A1 expression in both YAMC and Caco-2 cells. We also investigated butyrate (5 mM) enhancement of 0.001–10 nM TCDD effects in YAMC and Caco-2 cells (Fig. 2G) and show that butyrate comparably enhanced TCDD-induced *Cyp1a1* gene expression in both cell lines using high and low concentrations of TCDD.

### SCFAs enhance ligand-activated Ah-responsive genes

**Figure 3:**
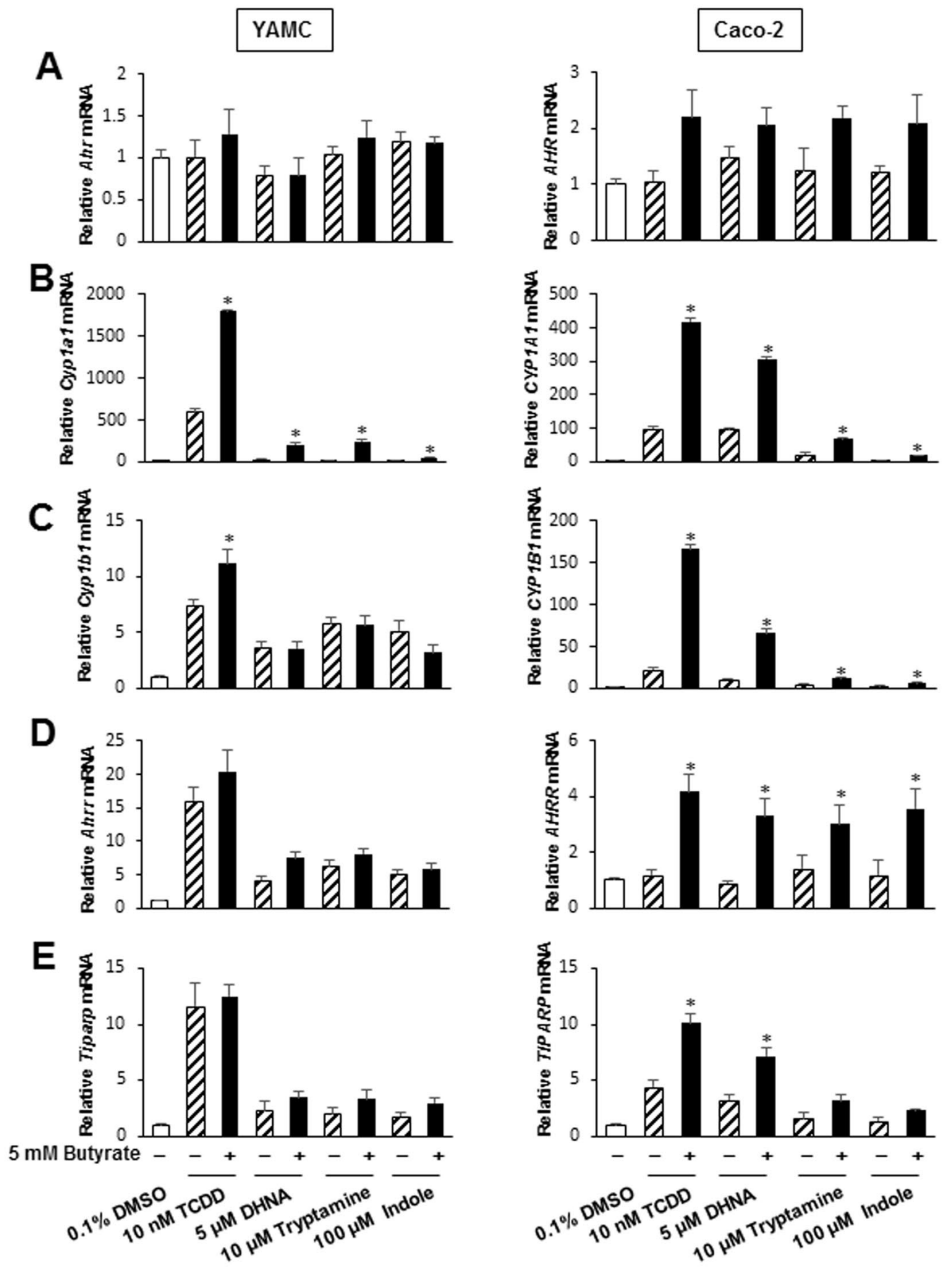
Butyrate enhances AhR ligand-induced gene expression in YAMC and Caco-2 cells. Cells were treated with DMSO, different AhR ligands alone or in combination with 5 mM butyrate and effects on expression of *AhR* (**A**), *Cyp1a1/CYP1A1* (**B**), *Cyp1b1/CYP1B1* (**C**), *Ahrr/AhRR* (**D**) and *Tiparp/TiPARP* (**E**) mRNA levels were determined by real time PCR. With the exception of *AhRR* mRNA, AhR ligands significantly induced all other Ah-responsive genes, and significant (p < 0.05) enhancement by butyrate is indicated (*). Results are expressed as means ± SE for at least 3 separate determinations for each treatment group.

Previous studies show that tryptophan metabolites tryptamine, indole and 1,4-dihydroxy-2-naphthoic acid (DHNA) are derived from microbiota and exhibit AhR agonist/antagonist activities in colon and other cell lines^30, 33–36^. Therefore, we investigated interactions of 5 mM butyrate with indole, tryptamine and DHNA and expression of AhR (Fig. 3A) and the Ah-responsive genes *Cyp1a1/CYP1A1* (Fig. 3B), *Cyp1b1/CYP1B1* (Fig. 3C), *Ahrr/AhRR* (Fig. 3D) and *Tiparp/TiPARP* (Fig. 3E) in YAMC and Caco-2 cells. Minimal interactions (<2-fold) were observed for AhR expression; however, the effects of butyrate on DHNA and the relative weak AhR agonists tryptamine and indole were comparable to those observed for TCDD (Fig. 2). Butyrate enhanced induction of Cyp1a1/CYP1A1 by indole, tryptamine and DHNA in YAMC and Caco-2 cells, and butyrate also enhanced induction of CYP1A1, AhRR, and TiPARP by the microbiota-derived metabolites only in Caco-2 cells. We also carried out an *in vivo* pilot study by treating C57BL/6 mice with butyrate (1 g/kg/d) and DHNA (20 mg/kg/d) for 3 days and observed minimal induction of Cyp1a1 or Cyp1a2 by the compounds alone but in combination, there was induction of Cyp1a1 (liver, colon) and Cyp1a2 (liver) (Suppl. Fig. S1).

**Figure 4:**
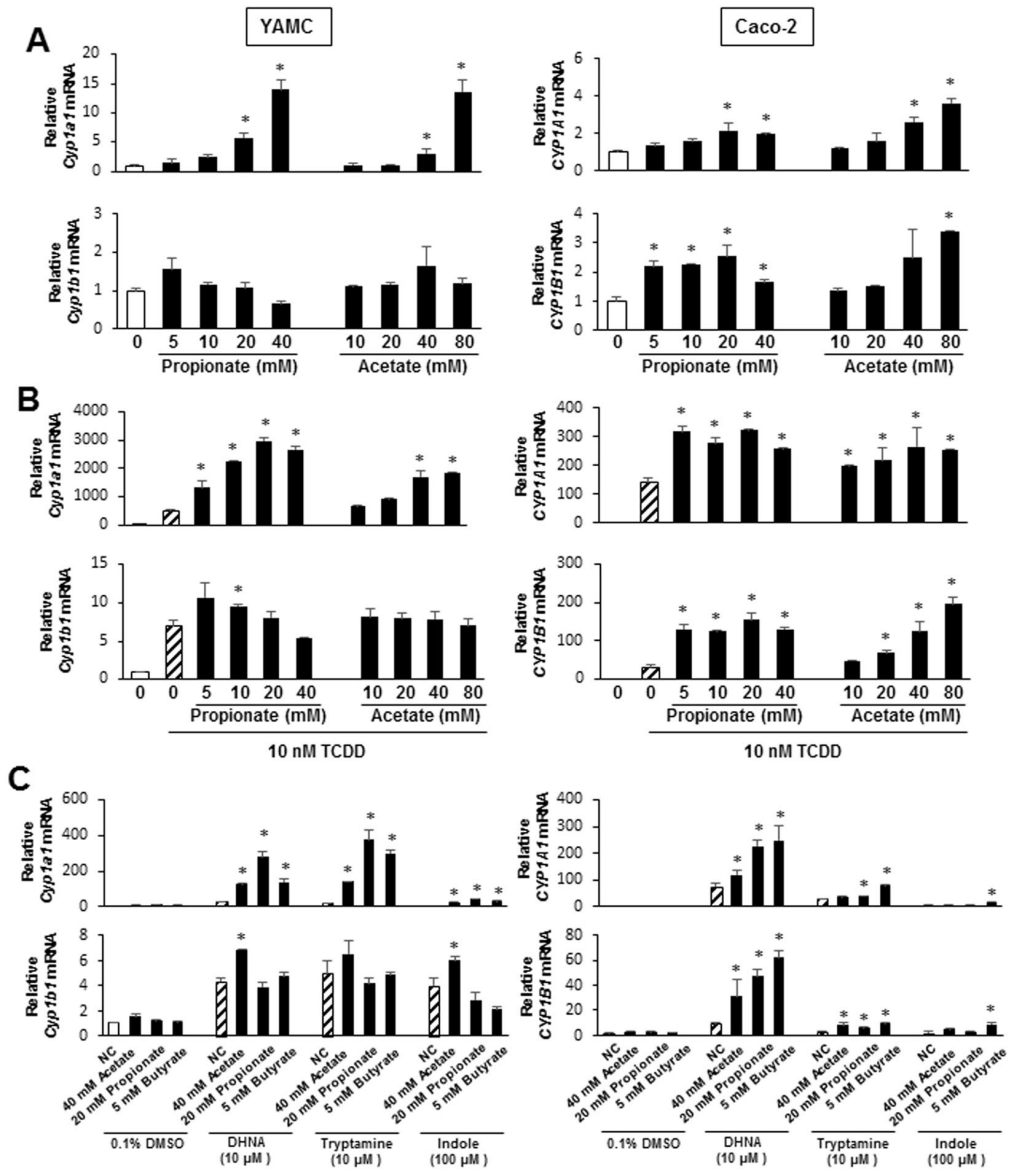
Acetate and propionate enhances *Cyp1a1/CYP1A1* expression in YAMC and Caco-2 cells. (**A**) Cells were treated with DMSO and different concentrations of acetate or propionate and expression of *Cyp1a1/CYP1A1* and *Cyp1b1/CYP1B1* mRNA levels was determined by real time PCR. (**B**) YAMA or Caco-2 cells were treated with DMSO, 10 nM TCDD alone and in combination with different concentrations of acetate or propionate, and expression of *Cyp1a1/CYP1A1* (**C**) and *Cyp1b1/CYP1B1* (**D**) mRNA levels was determined by real time PCR. (**C**) YAMC or Caco-2 cells were treated with DMSO, propionate and acetate alone and in combination with microbiota-derived AhR ligand, and *Cyp1a1/CYP1A1* and *Cyp1b1/CYP1B1* mRNA levels were determined by real time PCR. Significant (p < 0.05) induction of Cyps/CYPs by acetate or propionate alone and propionate/acetate-enhanced induction of Cyps/CYPs by AhR ligand is indicated (*). Results are expressed as means ± SE for at least 3 separate experiments for each treatment group.

Previous studies reported that the SCFAs butyrate and propionate, but not acetate, were HDAC inhibitors^26, 37, 38^ and therefore using *Cyp1a1* and *Cyp1b1* as Ah-responsive genes, we further investigated effects of propionate and acetate on Ah-responsive gene expression. Like butyrate, both propionate and acetate increased expression of *Cyp1a1/CYP1A1* in YAMC and Caco-2 cells and similar results were observed for expression of *Cyp1b1/CYP1B1* in both cell lines (Fig. 4A). Propionate and acetate also significantly enhanced TCDD-induced Cyp1a1 and CYP1A1 expression in YAMC and Caco-2 cells (Fig. 4B, top), respectively, and similar results were observed for butyrate (Fig. 2). In YAMC cells treated with TCDD, cotreatment with propionate or acetate had minimal effects on *Cyp1b1* mRNA levels, whereas both SCFAs significantly enhanced TCDD-induced CYP1B1 expression in Caco-2 cells (Fig. 4B, bottom). Results summarized in Fig. 4C show that propionate and acetate enhanced tryptophan-, indole- and DHNA-induced Cyp1a1 (YAMC and Caco-2 cells) and CYP1B1 (Caco-2 cells). Thus, acetate, propionate and butyrate, the major SCFAs generated by microbial fermentation of fiber in the gut, enhanced basal and AhR ligand-induced expression of Cyp1a1/CYP1A1in both Caco-2 and YAMC cells and enhancement of ligand-induced Cyp1b1/CYP1B1 was primarily observed in Caco-2 cells.

### HDAC inhibitors enhance expression of Ah-responsive genes

**Figure 5:**
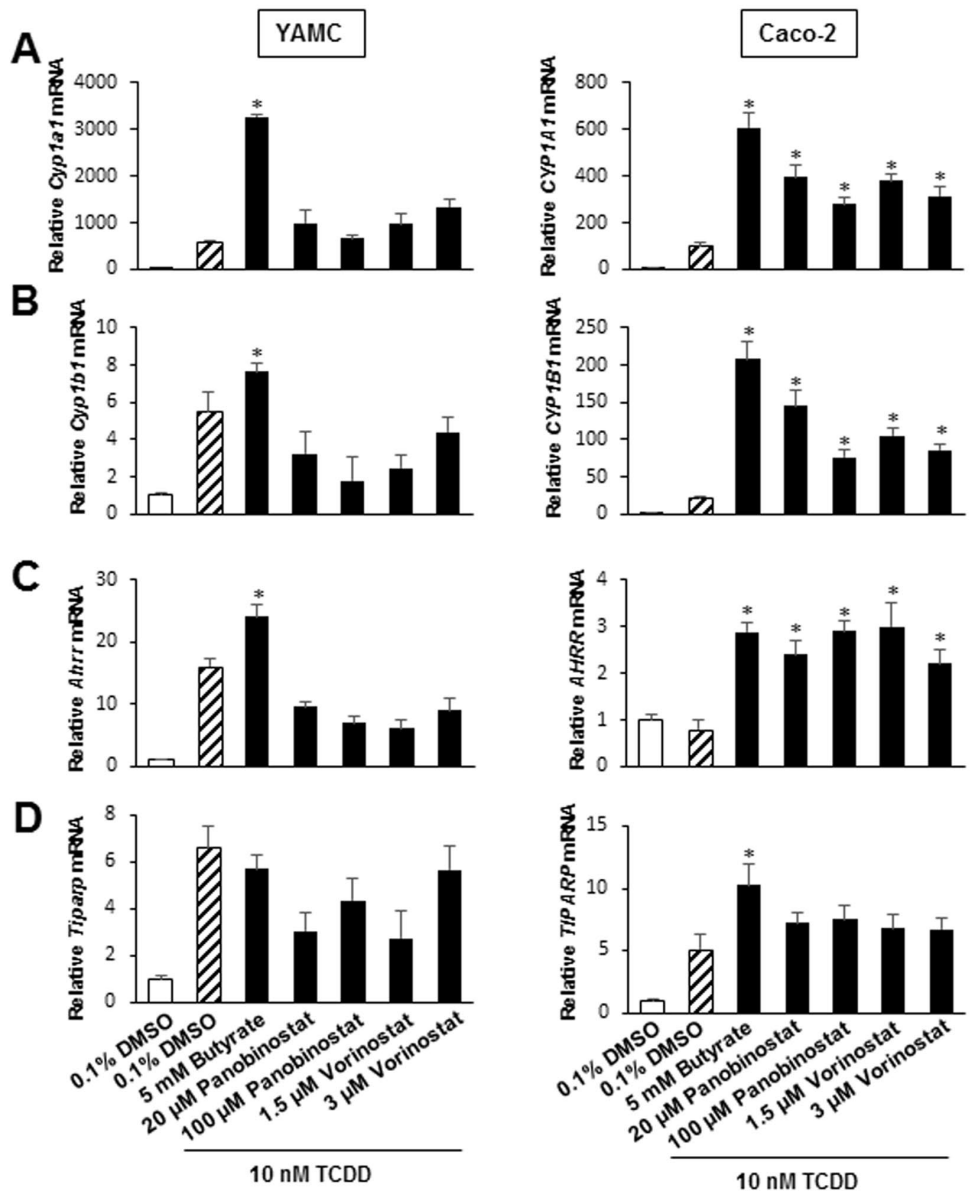
HDAC inhibitors enhance TCDD-induced gene expression in YAMC and Caco-2 cells. Cells were treated with DMSO alone, TCDD alone, and TCDD in combination with different HDAC inhibitors, and *Cyp1a1/CYP1A1* (**A**), *Cyp1b1/CYP1B1* (**B**), *Ahrr/AhRR* (**C**) and *Tiparp/TiPARP* (**D**) mRNA levels were determined by real time PCR. Significant (p < 0.05) enhancement of TCDD-induced gene expression by HDAC inhibitors is indicated (*). Results are expressed as means ± SE for 3 separate determinations for each treatment group.

We also investigated the effects of two synthetic HDAC inhibitors Panobinostat and Vorinostat on TCDD-induced Ah-responsive genes in YAMC and Caco-2 cells. In YAMC cells, Panobinostat and Vorinostat enhanced TCDD-induced *Cyp1a1/CYP1A1* gene expression (Fig. 5A) but at the concentration used they were less effective than butyrate. For TCDD-induced responses such as Cyp1b1/CYP1B1 (Fig. 5B), Ahrr (Fig. 5C) and Tiparp (Fig. 5D) where butyrate had minimal enhancing activity, similar results were observed for Panobinostat and Vorinastat, and the former compound decreased TCDD-induced Cyp1b1 and AhRR in YAMC cells. In contrast, like the SCFAs, Panobinostat and Vorinostat enhanced TCDD-induced CYP1B1, AhRR and TiPARP and with the exception of CYP1B1, the magnitude of enhanced responses for hydroxamic acid-derived synthetic HDAC inhibitors and SCFAs was similar.

### SCFAs enhance histone acetylation and recruitment of the AhR to Cyp1a1/CYP1A1 promoters

**Figure 6:**
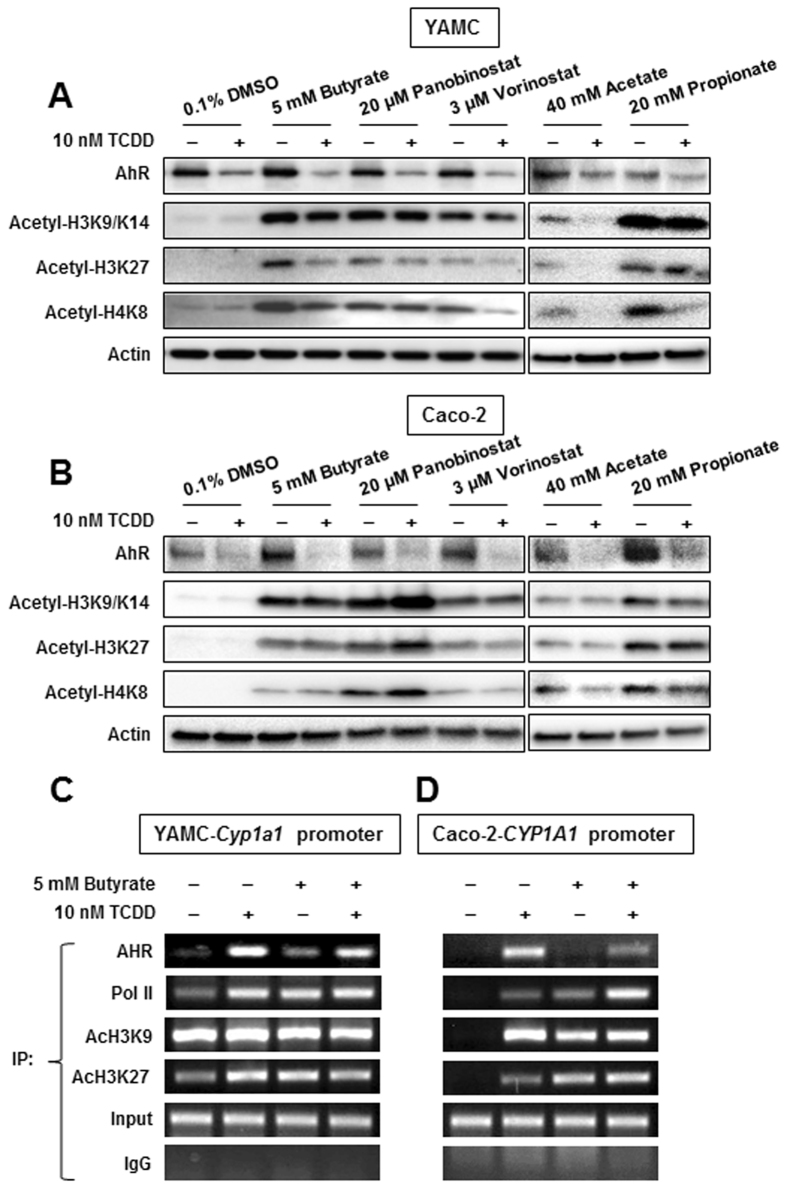
Effects of SCFAs and HDAC inhibitors on histone acetylation in ChIP assays. YAMC (**A**) and Caco-2 (**B**) cells were treated with DMSO, 10 nM TCDD alone or in combination with SCFAs and HDAC inhibitors for 24 hr, and whole cell lysates were analyzed by western blots. YAMC (**C**) and Caco-2 (**D**) cells were treated with DMSO, 10 nM TCDD and 5 mM butyrate alone or in combination for 4 hr, and ChIP assays were carried out as outlined in the Materials and Methods to detect interactions with the XRE-containing regions of the Cyp1a1 (mouse) and CYP1A1 (human) promoters.

After treating YAMC cells with 10 nM TCDD alone, butyrate, Panobinostat and Vorinostat alone, and in combination with TCDD for 18 hr, we examined expression of the AhR and acetylated histones (H3K9/K14, H3K27 and H4K8) by western blots. TCDD alone decreased AhR expression but did not affect histone acetylation in YAMC cells (Fig. 6A), whereas the HDAC inhibitors alone and in combination with TCDD increased acetylation of H3K9/K14, H3K27 and H4K8. Similar results were observed in Caco-2 cells (Fig. 6B), demonstrating that the HDAC inhibitors induced overall histone acetylation in both cell lines. We also investigated butyrate-TCDD/AhR interactions with an Ah-responsive (DRE-containing) region of the Cyp1a1 promoter in YAMC cells in a ChIP assay and observed that TCDD, butyrate and their combination induced recruitment of the AhR to the promoter and cotreatment did not further enhance AhR recruitment (Fig. 6C). Binding of pol II to the promoter was also enhanced by the different treatments and histone acetylation was observed in both the untreated and treated cells. In contrast, histone acetylation associated with untreated Caco-2 cells was not observed (Fig. 6D) but was induced by TCDD, butyrate and butyrate plus TCDD, and similar results were observed for treatment-related recruitment of the AhR and Pol II to the Ah-responsive region of the CYP1A1 promoter. Thus, there were major differences in histone acetylation of the Cyp1a1/CYP1A1 promoters in untreated YAMC and Caco-2 cells and this may account for the higher inducibility of this gene in YAMC cells.

## Discussion

The complex human gut microbiome plays a pivotal role in the health of the gastrointestinal tract and multiple distal organs and tissues^39^. These interactions are dependent on the diet and other factors that influence the composition of the microbiome and microbial metabolites that in turn directly affect the host through initial interactions with multiple targets on intestinal cells^39–41^. For example, humans on a plant-based vs. animal-based diet exhibited significant differences in bacterial taxonomic groups and this was particularly evident for the genus *Prevotella* which is induced by dietary fiber but is reduced in the animal-based diet^42^. Moreover, dietary-induced changes in the microbiome also resulted in changes in microbiota-derived metabolites in which SCFAs such as acetate and butyrate were higher in plant-based diets, whereas isovalerate and isobutyrate were higher in animal-based diets^42^. Plant-based diets are relatively high in fiber which induces commensal bacteria resulting in higher levels of SCFAs which in turn exhibit antiinflammatory activities^26–29^. Enhanced tryptophan metabolism by *Lactobacilli* induces formation of indole-derived Ah receptor ligands^19^ which also exhibit antiinflammatory activities and resistance to infection^18–25^. Moreover, tryptophan is degraded to form niacin, and both niacin and butyrate suppress intestinal inflammation by activation of the common receptor Gpr109a^43, 44^.

The effects of butyrate and other HDAC inhibitors on basal and ligand-induced Ah-responsive genes is highly variable; one study showed that butyrate enhanced an AhR-responsive gene promoter *in vitro* ^31^ and butyrate also enhanced expression of Ah-responsive genes in the mouse intestine^45^, whereas another study indicated that HDAC1 inhibition was insufficient to induce Cyp1a1^32^. The effects of HDAC inhibitors on ligand-induced CYP1A1 resulted in decreased expression in aerodigestive epithelial cells and increased expression in colon epithelial cells and fibroblasts^46–48^. This study extensively investigated AhR agonist-SCFA interactions on Ah-responsive gene expression *in vitro* using Caco-2 and YAMC cells which are responsive to both TCDD, DHNA and tryptophan metabolites^30, 33, 34^.

Among the SCFAs butyrate has been extensively identified as an HDAC inhibitor. Thus, we initially examined the effects of butyrate alone and in combination with TCDD and three microbiota-derived AhR ligands, indole, tryptamine and DHNA on expression of four Ah-responsive genes (*Cyp1a1, Cyp1b1, AhRR* and *TiPARP*). Butyrate induced *Cyp1a1/CYP1A1* and *Cyp1b1/CYP1B1* mRNA levels in both YAMC and Caco-2 cells (Fig. 1) and the latter response was accompanied by enhanced histone acetylation (H3K9/14, H3K27 and H4K8) in both cell lines (Fig. 6). Moreover, in ChIP assays, we also observed that treatment with butyrate recruited the AhR and pol II to the Cyp1a1 promoter in both cell lines and enhanced H3K47Ac and H3K9Ac on the CYP1A1 promoter only in Caco-2 cells (Fig. 6A–D), whereas the histone marks were observed on the Cyp1a1 promoter in untreated YAMC cells. These results are consistent with an induction (Cyp1a1) response by butyrate due to its activity as an HDAC inhibitor and similar results were observed for hydroxamic acid-derived HDAC inhibitors Vorinostat and Panobinostat (Fig. 5). Butyrate also significantly enhanced *AhRR* and *TiPARP* gene expression in Caco-2 cells but the magnitude of these responses (1.5- to 3-fold) were lower than observed for Cyp1a1. The effects of butyrate alone on Ah-responsive genes in YAMC and Caco-2 cells was also observed for propionate, and this is consistent with previous studies showing that like butyrate, propionate is also an HDAC inhibitor^26, 37, 38^. The highest concentration SCFA produced by microorganisms in the gut is usually acetate^26^ and in this study, we observed that acetate concentrations >20 mM also enhanced expression of Ah-responsive genes in YAMC and Caco-2 cells (Fig. 4). Acetate also enhanced overall histone acetylation in these cell lines (Fig. 6), demonstrating for the first time that acetate is also an HDAC inhibitor and thus, contributes to the overall HDAC inhibitory activity of SCFAs.

We also observed that the effects of all three SCFAs on AhR ligand-induced gene expression were similar for TCDD, tryptamine, indole and DHNA but were both cell context- and gene-dependent. AhR ligand-induced Cyp1a1/CYP1A1 was enhanced by SCFAs in both YAMC and Caco-2 and similar results were observed for the hydroxamic acid-derived HDAC inhibitors Panobinostat and Vorinostat. The HDAC inhibitors enhanced AhR ligand-mediated induction of CYP1B1, AhRR and TiPARP in Caco-2 cells, whereas in YAMC cells, the enhancement was minimal to non-detectable and this was similar to that observed for SCFAs.

The reasons for the gene- and cell-specific interactions of SCFAs and AhR ligands and their impacts on intestinal health are unknown and are currently under investigation. However, since recent reports indicate that both the AhR and Cyp1a1 are necessary for maintaining health-promoting levels of microbiota-derived natural AhR ligands^49–51^, it is possible that SCFAs also play a role in maintaining intestinal AhR and enhanced Cyp1a1 activity *in vivo*. In a pilot study, we showed that treatment of C57BL/6 mice with 1 g/kg/d of butyrate and 20 mg/kg/d of DHNA as previously described^45, 52^ did not significantly induce liver or colon Cyp1a1 or Cyp1a2 (Suppl. Fig. S1). The reason for these differences are unclear; however, it should be noted the magnitude of the reported induction responses was small^45, 52^. In contrast, we observed induction of Cyp1a1 in the liver and colon in animals cotreated with DHNA plus butyrate, and over 50-fold induction was observed in liver with a large standard error due to mouse variability. DHNA plus butyrate also significantly induced Cyp1a2 in liver but not colon and this is consistent with a previous report indicating that Cyp1a2 was not inducible in the mouse colon^53^. We are currently refining methods for enhancing SCFA production and future studies will investigate the role of SCFAs on AhR ligand-dependent activation of IL-22 and Tregs and inhibition of intestinal inflammation in mouse models.

## Electronic supplementary material

Supplemental Material
