# Supplementary material for: Short Chain Fatty Acids Enhance Aryl Hydrocarbon (Ah) Responsiveness in Mouse Colonocytes and Caco-2 Human Colon Cancer Cells
Source: Sci Rep. 2017 Aug 31;7:10163. doi: 10.1038/s41598-017-10824-x (PMC5579248; doi:10.1038/s41598-017-10824-x)
Supplement: Supplementary file 1 — Supplemental Material [file 41598_2017_10824_MOESM1_ESM.pdf]

SUPPLEMENTAL MATERIAL

Short Chain Fatty Acids Enhance Aryl Hydrocarbon (Ah) Responsiveness in Mouse Colonocytes and Caco-2 Human Colon Cancer Cells

Un-Ho Jin[1]*, Yating Cheng[1]*, Hyejin Park[1], Laurie A. Davidson[2], Evelyn S. Callaway[2], Robert S. Chapkin[2], Arul Jayaraman[3], Andrew Asante[4], Clinton Allred[2], Evelyn A. Weaver[5], and Stephen Safe[1]

Supplementary Table S1.

Mouse primers

Name	Forward Primer	Reverse Primer
Tbp	GAACAATCCAGACTAGCAGCA	GGGAACTTCACATCACAGCTC
Cyp1a1	ATCCAAGGCAGAATACGGTG	TCCACTCCATCTTCCGACTT
Cyp1b1	GGATATCAGCCACGACGAAT	ATTATCTGGGCAAAGCAACG
Tiparp	GCCAGACTGTGTAGTACAGCC	GGGTTCCAGTTCCCAATCTTTT
Ahrr	ACATACGCCGGTAGGAAGAGA	GGTCCAGCTCTGTATTGAGGC
Ahr	GCCCTTCCCGCAAGATGTTAT	GCTGACGCTGAGCCTAAGAAC

Human Primers

Name	Forward Primer	Reverse Primer
TBP	GATCAGAACAACAGCCTGCC	TTCTGAATAGGCTGTGGGGT
CYP1A1	GACCACAACCACCAAGAAC	AGCGAAGAATAGGGATGAAG
CYP1B1	CACTGACATCTTCGGCG	ACCTGATCCAATTCTGCCTG
TIPARP	AGAACGAGTGGTTCCAATCCA	TGGGTGCAAAAGATCAGTCTG
AHRR	CAGTGACTGTCAGGTCGATCA	GCCTCTGTTTCTGCAGGG
AHR	TCAGTTCTTAGGCTCAGCGTC	AGTTATCCTGGCCTCCGTTT

Supplement Figure S1

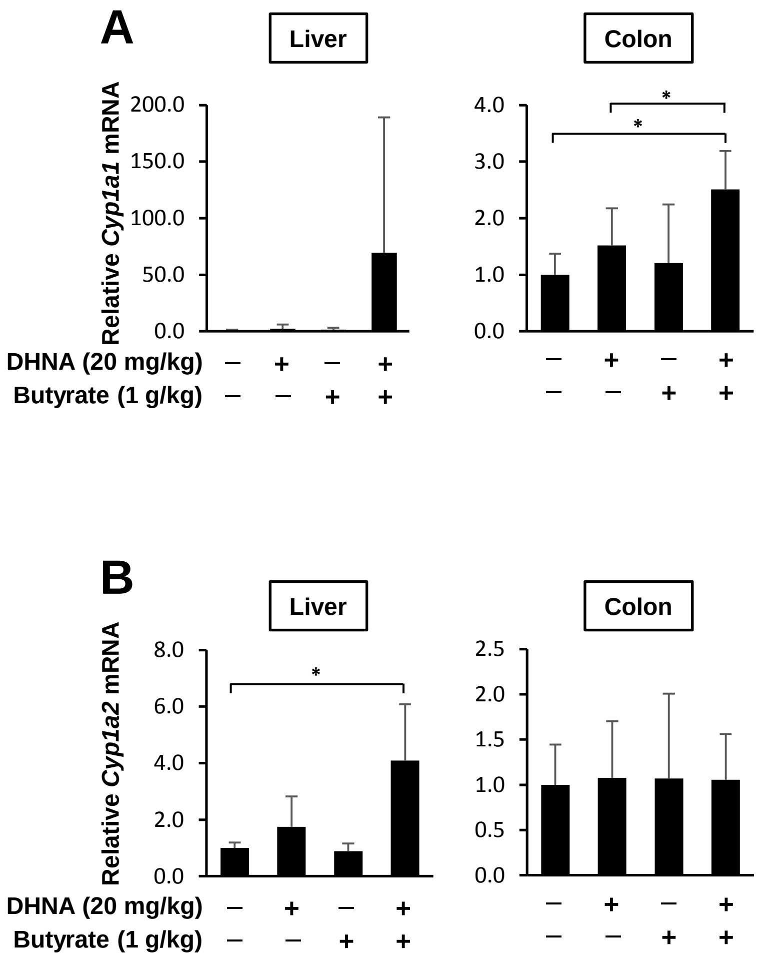

Supplemental Figure S1. Butyrate-DHNA interactions *in vivo*. Mice (5/group) were administered butyrate (1 g/kg/d) and DHNA (20 mg/kg/d) alone and in combination for 3 consecutive days, and 6 hr after the final dose, mice were sacrificed and liver and colon *Cyp1a1* (A) and *Cyp1a2* (B) mRNA levels were determined by real time PCR. Results are expressed as means ± SE, and significance ($p<0.05$) is indicated (*).
